# Une mélanonychie suspecte révélant un syndrome de Laugier Hunziker

**DOI:** 10.11604/pamj.2015.22.291.8203

**Published:** 2015-11-24

**Authors:** Ramli Inssaf, Senouci Karima

**Affiliations:** 1 Service de Dermatologie et Vénérologie, CHU Ibn Sina, Université Mohammed V, Rabat Maroc

**Keywords:** Mélanonychie, maladie de Laugier Hunziker, mélanome, melanonychia, Laugier Hunziker disease, melanoma

## Image en medicine

Le syndrome de Laugier Hunziker (SLH) est une affection rare, d’étiologie inconnue, décrite initialement en 1970 par Laugier et Hunziker. Elle touche les adultes de phototype clair avec une prédominance féminine. Ce syndrome est caractérisépar la présence des macules lenticulaires, bien limitées, de couleur variable, siégeant au niveau des lèvres et de la cavité buccale. L'atteinte unguéale se voit chez plus de 60% des cas. Trois types de pigmentation unguéale ont été décrits. Le signe de Hutchinson peut être positif posant le problème de diagnostic différentiel avec un mélanome unguéal, surtout lorsqu'il s'agit d'une atteinte monodactylique. Le SLH fait partie des pigmentations mélaniques lenticulaires acquises et bénignes. Aucun cas de transformation maligne n'a été rapporté. Le traitement est purement esthétique, le laser Nd-Yag reste la technique de choix,le risque de récidive n'est pas négligeable.Nous rapportons le cas d'une SLH révélée par une mélanonychie longitudinale suspecte. L’étude dermoscopique et histologique de l'ongle ont permis d’éliminer un mélanome. L'examen cutanéo-muqueux a objectivé des macules lenticulaires des lèvres, du menton et de la région péri-anale. Une abstention thérapeutique a été décidée associée à une surveillance régulière de la patiente.

**Figure 1:**
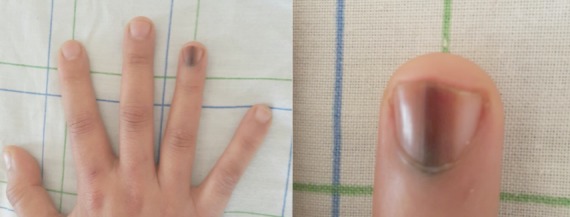
Mélanonychie hétérogène avec un signe de Hutchinson positif

